# *ent*-(15*S*)-Pimar-8(14)-ene-15,16-diol

**DOI:** 10.1107/S1600536812002565

**Published:** 2012-01-25

**Authors:** Hoong-Kun Fun, Suchada Chantrapromma, Charoen Pakhathirathien, Chatchanok Karalai, Kan Chantrapromma

**Affiliations:** a X-ray Crystallography Unit, School of Physics, Universiti Sains Malaysia, 11800 USM, Penang, Malaysia; b Crystal Materials Research Unit, Department of Chemistry, Faculty of Science, Prince of Songkla University, Hat-Yai, Songkhla 90112, Thailand; c Department of Chemistry, Faculty of Science, Prince of Songkla University, Hat-Yai, Songkhla 90112, Thailand; d Research Unit of Natural Products Utilization, Walailak University, Thasala, Nakhon Si Thammarat 80160, Thailand

## Abstract

The title compound {systematic name: (*S*)-1-[(2*S*,4a*R*,8a*R*)-2,4b,8,8-tetra­methyl-2,3,4,4a,4b,5,6,7,8,8a,9,10-dodeca­hydro­phenanthren-2-yl]ethane-1,2-diol}, C_20_H_34_O_2_, is an *ent*-pimarane diterpenoid which was isolated from the stem bark of *Ceriops tagal*. In the asymmetric unit, there are two crystallographically independent mol­ecules, which are conformationally almost identical. In each mol­ecule, the two cyclo­hexane rings of the fused three-ring system adopt chair conformations, while the cyclo­hexene ring is in an envelope conformation, with the methylene C atom next to the side chain as the flap atom. In the crystal, mol­ecules are stacked in columns along the *b* axis through O—H⋯O hydrogen bonds.

## Related literature

For ring conformations, see: Cremer & Pople (1975 ▶). For standard bond lengths, see: Allen *et al.* (1987 ▶). For bioactive compounds from *Ceriops tagal* and their activities, see: Bamroongrugsa (1999 ▶); Chacha (2011 ▶); Pakhathirathien *et al.* (2005 ▶); Zhang *et al.* (2005 ▶). For related structures, see: Chantrapromma *et al.* (2007 ▶); Fun *et al.* (2006 ▶, 2010 ▶).
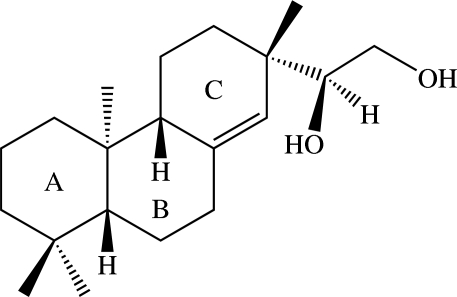


## Experimental

### 

#### Crystal data

C_20_H_34_O_2_
*M* *_r_* = 306.47
Monoclinic, 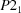

*a* = 11.5129 (2) Å
*b* = 7.0934 (1) Å
*c* = 22.3946 (4) Å
β = 96.750 (1)°
*V* = 1816.19 (5) Å^3^
*Z* = 4
Mo *K*α radiation
μ = 0.07 mm^−1^
*T* = 273 K
0.56 × 0.34 × 0.25 mm

#### Data collection

Bruker SMART APEX2 CCD area-detector diffractometer
Absorption correction: multi-scan (*SADABS*; Bruker, 2005 ▶) *T*_min_ = 0.962, *T*_max_ = 0.983
28246 measured reflections
5205 independent reflections
4504 reflections with *I* > 2σ(*I*)
*R*_int_ = 0.031

#### Refinement

*R*[*F* ^2^ > 2σ(*F* ^2^)] = 0.041
*wR*(*F* ^2^) = 0.106
*S* = 1.03
5205 reflections
421 parameters
1 restraint
H atoms treated by a mixture of independent and constrained refinement
Δρ_max_ = 0.17 e Å^−3^
Δρ_min_ = −0.16 e Å^−3^

### 

Data collection: *APEX2* (Bruker, 2005 ▶); cell refinement: *SAINT* (Bruker, 2005 ▶); data reduction: *SAINT*; program(s) used to solve structure: *SHELXTL* (Sheldrick, 2008 ▶); program(s) used to refine structure: *SHELXTL*; molecular graphics: *SHELXTL*; software used to prepare material for publication: *SHELXTL* and *PLATON* (Spek, 2009 ▶).

## Supplementary Material

Crystal structure: contains datablock(s) global, I. DOI: 10.1107/S1600536812002565/is5054sup1.cif

Structure factors: contains datablock(s) I. DOI: 10.1107/S1600536812002565/is5054Isup2.hkl

Supplementary material file. DOI: 10.1107/S1600536812002565/is5054Isup3.cml

Additional supplementary materials:  crystallographic information; 3D view; checkCIF report

## Figures and Tables

**Table 1:** Hydrogen-bond geometry (Å, °)

*D*—H⋯*A*	*D*—H	H⋯*A*	*D*⋯*A*	*D*—H⋯*A*
O1*A*—H1*OA*⋯O1*B*^i^	0.78 (3)	2.06 (3)	2.839 (2)	174 (3)
O2*A*—H1*OB*⋯O1*B*^i^	0.84 (4)	2.14 (4)	2.931 (2)	158 (3)
O1*B*—H1*OC*⋯O2*B*^ii^	0.76 (2)	1.96 (2)	2.719 (2)	178 (3)
O2*B*—H1*OD*⋯O1*A*^iii^	0.77 (3)	2.13 (3)	2.795 (3)	145 (3)

Symmetry codes: (i) 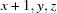; (ii) 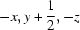; (iii) 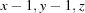.

**Fig. 1.**
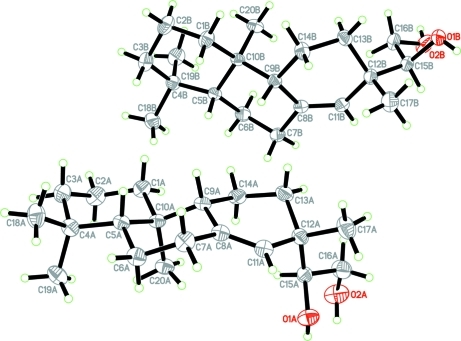
The asymmetric unit of the title compound, showing 30% probability displacement ellipsoids and the atom-numbering scheme.

**Fig. 2.**
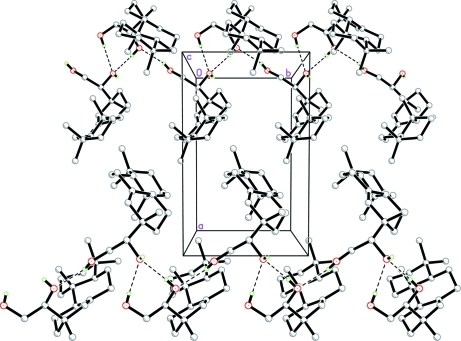
A packing diagram of the title compound viewed along the c axis, showing screw chains running along the [0 1 0] direction. For clarity, H atoms not involved in hydrogen bonds were omitted. Hydrogen bonds are shown as dashed lines.

## supplementary crystallographic information

### Comment

Diterpenoids and triterpenoids are the main secondary metabolites of *Ceriops tagal* (Chacha, 2011; Pakhathirathien *et al.*, 2005; Zhang *et al.*, 2005). The decoction of this mangrove plant was used as substitute for quinine as treatment for Malaria and the bark has been used for the treatment of infected wounds (Bamroongrugsa, 1999). During the course of our studies on the chemical constituents and bioactive compounds from Thai medicinal plants, the title compound (I) which is a new *ent* pimarane diterpenoid, named as "Ceriotagalsin A", was isolated from the stem bark of *C. tagal*. We have also previously reported the crystal structures of diterpenoids isolated from the same plant (Chantrapromma *et al.*, 2007; Fun *et al.*, 2006, 2010). (I) was tested for antimalarial activity and found to be inactive. We herein report the crystal structure of (I).

The title compound (I) (Fig. 1) crystallized out with two crystallographically independent molecules *A* and *B* per asymmetric unit with slight differences in bond angles. The molecule of (I) contains a fused three-ring system *A/B/C* (Fig. 1). The *A*/*B* ring junction is *trans*-fused. In both molecules, the cyclohexane ring *A* and *B* have standard chair conformations (Cremer & Pople 1975). The cyclohexene ring *C* adopts an envelope conformation with the puckered C13 atom having the maximum deviation of 0.335 (2) Å and puckering parameters Q = 0.481 (2) Å, θ = 125.7 (2)° and φ = 347.9 (3)° in molecule *A* [the corresponding values are -0.329 (2) Å, Q = 0.465 (2) Å, θ = 54.8 (2)° and φ = 177.1 (3)° in molecule *B*]. The 1,2-hydroxyethyl is bisectionally attached to the cyclohexene ring at atom C12 with the torsion angle C11—C12—C15—O1 = -47.6 (2)° and C12—C15—C16—O2 = -162.28 (18)° in molecule *A* [the corresponding values are -176.96 (13) and -161.55 (17)° in molecule *B*]. The C8═C11 bond length [1.326 (3) Å in molecule *A* and 1.328 (2) Å in molecule *B*] and bond angles around atoms C8 and C11 indicate the *sp*^2^ hybridization for these atoms. The bond distances are of normal values (Allen *et al.*, 1987) and are comparable with the related structures (Chantrapromma *et al.*, 2007; Fun *et al.*, 2006, 2010).

In the crystal structure (Fig. 2), the molecules are linked into one dimensional screw chains along the [0 1 0] direction. The crystal packing is stabilized by intermolecular O—H···O hydrogen bonds (Table 1).

### Experimental

The air-dried and crushed stem bark of *C. tagal* (4.8 kg) were extracted with methylene chloride and then concentrated *in vacuo* to give a residue (17.4 g). This residue was subjected to quick column chromatography over silica gel using solvents of increasing polarity from hexane through 50% acetone/hexane. The eluates were collected and combined based on TLC to give 20 fractions (F1—F20). Fraction F14 was further purified by repeated quick column chromatography with CH_2_Cl_2_/acetone (9:1 *v*/*v*) yielding the title compound (30.1 mg). Colorless needle-shaped single crystals of the title compound suitable for *x*-ray structure determination were recrystallized from acetone after several days (m.p. 377–378 K).

### Refinement

Hydroxy H atoms were located from the difference maps and freely refined [refined O—H distances 0.76 (2)–0.84 (4) Å]. The remaining H atoms were placed in calculated positions with C—H = 0.93 Å for aromatic, 0.98 Å for CH, 0.97 Å for CH_2_ and 0.96 Å for CH_3_ atoms. The *U*_iso_(H) values were constrained to be 1.5*U*_eq_ of the carrier atom for methyl H atoms and 1.2*U*_eq_ for the remaining H atoms. A rotating group model was used for the methyl groups. A total of 3692 Friedel pairs were merged before final refinement as there is no large anomalous dispersion for the determination of the absolute configuration.

### Crystal data

**Table:**

C_20_H_34_O_2_	*F*(000) = 680
*M**_r_* = 306.47	*D*_x_ = 1.121 Mg m^−^^3^
Monoclinic, *P*2_1_	Melting point = 377–378 K
Hall symbol: P 2yb	Mo *K*α radiation, λ = 0.71073 Å
*a* = 11.5129 (2) Å	Cell parameters from 5205 reflections
*b* = 7.0934 (1) Å	θ = 1.8–29.0°
*c* = 22.3946 (4) Å	µ = 0.07 mm^−^^1^
β = 96.750 (1)°	*T* = 273 K
*V* = 1816.19 (5) Å^3^	Needle, colorless
*Z* = 4	0.56 × 0.34 × 0.25 mm

### Data collection

**Table:**

Bruker SMART APEX2 CCD area-detector diffractometer	5205 independent reflections
Radiation source: fine-focus sealed tube	4504 reflections with *I* > 2σ(*I*)
graphite	*R*_int_ = 0.031
Detector resolution: 8.33 pixels mm^-1^	θ_max_ = 29.0°, θ_min_ = 1.8°
ω scans	*h* = −15→15
Absorption correction: multi-scan (*SADABS*; Bruker, 2005)	*k* = −9→9
*T*_min_ = 0.962, *T*_max_ = 0.983	*l* = −30→30
28246 measured reflections	

### Refinement

**Table:**

Refinement on *F*^2^	Primary atom site location: structure-invariant direct methods
Least-squares matrix: full	Secondary atom site location: difference Fourier map
*R*[*F*^2^ > 2σ(*F*^2^)] = 0.041	Hydrogen site location: inferred from neighbouring sites
*wR*(*F*^2^) = 0.106	H atoms treated by a mixture of independent and constrained refinement
*S* = 1.03	*w* = 1/[σ^2^(*F*_o_^2^) + (0.0565*P*)^2^ + 0.1315*P*] where *P* = (*F*_o_^2^ + 2*F*_c_^2^)/3
5205 reflections	(Δ/σ)_max_ = 0.001
421 parameters	Δρ_max_ = 0.17 e Å^−^^3^
1 restraint	Δρ_min_ = −0.16 e Å^−^^3^

### Special details

**Table:**

Geometry. All e.s.d.'s (except the e.s.d. in the dihedral angle between two l.s. planes) are estimated using the full covariance matrix. The cell e.s.d.'s are taken into account individually in the estimation of e.s.d.'s in distances, angles and torsion angles; correlations between e.s.d.'s in cell parameters are only used when they are defined by crystal symmetry. An approximate (isotropic) treatment of cell e.s.d.'s is used for estimating e.s.d.'s involving l.s. planes.
Refinement. Refinement of *F*^2^ against ALL reflections. The weighted *R*-factor *wR* and goodness of fit *S* are based on *F*^2^, conventional *R*-factors *R* are based on *F*, with *F* set to zero for negative *F*^2^. The threshold expression of *F*^2^ > σ(*F*^2^) is used only for calculating *R*-factors(gt) *etc*. and is not relevant to the choice of reflections for refinement. *R*-factors based on *F*^2^ are statistically about twice as large as those based on *F*, and *R*- factors based on ALL data will be even larger.

### Fractional atomic coordinates and isotropic or equivalent isotropic displacement parameters (Å^2^)

**Table:**

	*x*	*y*	*z*	*U*_iso_*/*U*_eq_	
O1A	0.81903 (14)	0.4262 (2)	0.07420 (7)	0.0536 (4)	
H1OA	0.866 (2)	0.351 (4)	0.0679 (11)	0.058 (8)*	
O2A	0.73660 (18)	0.0232 (3)	0.05561 (9)	0.0749 (5)	
H1OB	0.799 (3)	0.052 (6)	0.0421 (14)	0.098 (11)*	
C1A	0.72183 (17)	0.3489 (3)	0.35428 (8)	0.0472 (4)	
H1AA	0.6477	0.4036	0.3617	0.057*	
H1AB	0.7059	0.2259	0.3362	0.057*	
C2A	0.7980 (2)	0.3239 (3)	0.41415 (10)	0.0562 (5)	
H2AA	0.7571	0.2468	0.4407	0.067*	
H2AB	0.8698	0.2595	0.4076	0.067*	
C3A	0.82712 (19)	0.5136 (4)	0.44337 (9)	0.0527 (5)	
H3AA	0.8753	0.4938	0.4814	0.063*	
H3AB	0.7552	0.5737	0.4519	0.063*	
C4A	0.89149 (16)	0.6458 (3)	0.40430 (9)	0.0447 (4)	
C5A	0.81852 (14)	0.6626 (3)	0.34104 (8)	0.0361 (4)	
H5AA	0.7460	0.7256	0.3488	0.043*	
C6A	0.87199 (17)	0.7930 (3)	0.29730 (9)	0.0455 (4)	
H6AA	0.8976	0.9089	0.3177	0.055*	
H6AB	0.9398	0.7327	0.2837	0.055*	
C7A	0.78277 (18)	0.8383 (3)	0.24320 (9)	0.0465 (4)	
H7AA	0.7224	0.9188	0.2562	0.056*	
H7AB	0.8214	0.9080	0.2139	0.056*	
C8A	0.72631 (15)	0.6656 (3)	0.21346 (8)	0.0390 (4)	
C9A	0.68153 (13)	0.5262 (3)	0.25669 (8)	0.0364 (4)	
H9AA	0.6197	0.5917	0.2751	0.044*	
C10A	0.77758 (14)	0.4746 (3)	0.30945 (8)	0.0352 (4)	
C11A	0.71179 (15)	0.6440 (3)	0.15422 (9)	0.0427 (4)	
H11A	0.7472	0.7324	0.1316	0.051*	
C12A	0.64268 (14)	0.4882 (3)	0.12021 (8)	0.0431 (4)	
C13A	0.56019 (15)	0.4038 (4)	0.16287 (9)	0.0495 (5)	
H13A	0.4986	0.4938	0.1678	0.059*	
H13B	0.5238	0.2910	0.1447	0.059*	
C14A	0.62334 (16)	0.3544 (3)	0.22433 (8)	0.0455 (4)	
H14A	0.6825	0.2600	0.2196	0.055*	
H14B	0.5678	0.3003	0.2489	0.055*	
C15A	0.72612 (15)	0.3356 (3)	0.10103 (8)	0.0409 (4)	
H15A	0.7609	0.2706	0.1374	0.049*	
C16A	0.6687 (2)	0.1888 (4)	0.05847 (12)	0.0647 (7)	
H16A	0.5937	0.1551	0.0711	0.078*	
H16B	0.6541	0.2430	0.0185	0.078*	
C17A	0.5698 (2)	0.5755 (4)	0.06543 (11)	0.0661 (7)	
H17A	0.5255	0.6797	0.0781	0.099*	
H17B	0.5175	0.4823	0.0464	0.099*	
H17C	0.6209	0.6192	0.0374	0.099*	
C18A	0.8966 (2)	0.8406 (4)	0.43453 (11)	0.0639 (6)	
H18A	0.9267	0.8278	0.4762	0.096*	
H18B	0.8194	0.8936	0.4314	0.096*	
H18C	0.9468	0.9220	0.4149	0.096*	
C19A	1.01865 (16)	0.5797 (4)	0.40298 (11)	0.0591 (6)	
H19A	1.0628	0.6036	0.4413	0.089*	
H19B	1.0529	0.6472	0.3723	0.089*	
H19C	1.0195	0.4470	0.3945	0.089*	
C20A	0.87771 (16)	0.3679 (3)	0.28404 (9)	0.0456 (4)	
H20A	0.8525	0.2422	0.2731	0.068*	
H20B	0.9444	0.3623	0.3140	0.068*	
H20C	0.8985	0.4326	0.2492	0.068*	
O1B	−0.02356 (11)	0.1434 (3)	0.04385 (6)	0.0461 (3)	
H1OC	−0.008 (2)	0.172 (4)	0.0131 (10)	0.048 (6)*	
O2B	−0.03925 (16)	−0.2575 (3)	0.06519 (7)	0.0609 (5)	
H1OD	−0.082 (2)	−0.320 (5)	0.0804 (12)	0.068 (9)*	
C1B	0.27744 (18)	0.3103 (3)	0.34629 (8)	0.0460 (4)	
H1BA	0.3426	0.3906	0.3392	0.055*	
H1BB	0.2060	0.3815	0.3358	0.055*	
C2B	0.2877 (2)	0.2604 (4)	0.41295 (9)	0.0555 (5)	
H2BA	0.2885	0.3750	0.4366	0.067*	
H2BB	0.2206	0.1860	0.4210	0.067*	
C3B	0.39891 (19)	0.1496 (4)	0.43098 (9)	0.0512 (5)	
H3BA	0.4028	0.1179	0.4733	0.061*	
H3BB	0.4655	0.2294	0.4259	0.061*	
C4B	0.40886 (15)	−0.0322 (3)	0.39499 (8)	0.0413 (4)	
C5B	0.38623 (13)	0.0151 (3)	0.32653 (7)	0.0346 (4)	
H5BA	0.4526	0.0944	0.3190	0.041*	
C6B	0.39381 (17)	−0.1555 (3)	0.28551 (8)	0.0463 (4)	
H6BA	0.3238	−0.2318	0.2854	0.056*	
H6BB	0.4606	−0.2324	0.3005	0.056*	
C7B	0.40626 (16)	−0.0902 (4)	0.22146 (8)	0.0497 (5)	
H7BA	0.4814	−0.0287	0.2209	0.060*	
H7BB	0.4044	−0.1992	0.1952	0.060*	
C8B	0.31058 (14)	0.0438 (3)	0.19795 (8)	0.0387 (4)	
C9B	0.28974 (14)	0.2057 (3)	0.23949 (7)	0.0352 (4)	
H9BA	0.3623	0.2793	0.2433	0.042*	
C10B	0.27621 (13)	0.1366 (3)	0.30492 (7)	0.0325 (3)	
C11B	0.25326 (14)	0.0238 (3)	0.14331 (8)	0.0425 (4)	
H11B	0.2741	−0.0777	0.1205	0.051*	
C12B	0.15718 (15)	0.1514 (3)	0.11489 (7)	0.0399 (4)	
C13B	0.10155 (15)	0.2509 (3)	0.16542 (8)	0.0441 (4)	
H13C	0.0479	0.3470	0.1481	0.053*	
H13D	0.0570	0.1600	0.1857	0.053*	
C14B	0.19380 (16)	0.3423 (3)	0.21132 (8)	0.0447 (4)	
H14C	0.1548	0.3970	0.2433	0.054*	
H14D	0.2309	0.4442	0.1918	0.054*	
C15B	0.06752 (14)	0.0294 (3)	0.07492 (7)	0.0395 (4)	
H15B	0.1092	−0.0318	0.0445	0.047*	
C16B	0.00925 (17)	−0.1240 (3)	0.10784 (8)	0.0461 (5)	
H16C	0.0662	−0.1844	0.1371	0.055*	
H16D	−0.0517	−0.0702	0.1290	0.055*	
C17B	0.20945 (19)	0.2949 (4)	0.07418 (9)	0.0586 (6)	
H17D	0.2701	0.3653	0.0974	0.088*	
H17E	0.1493	0.3795	0.0573	0.088*	
H17F	0.2417	0.2297	0.0424	0.088*	
C18B	0.53511 (17)	−0.1040 (4)	0.40919 (9)	0.0621 (7)	
H18D	0.5566	−0.1041	0.4519	0.093*	
H18E	0.5870	−0.0228	0.3905	0.093*	
H18F	0.5405	−0.2298	0.3940	0.093*	
C19B	0.3277 (2)	−0.1827 (4)	0.41623 (10)	0.0555 (5)	
H19D	0.3560	−0.2201	0.4565	0.083*	
H19E	0.3261	−0.2901	0.3901	0.083*	
H19F	0.2502	−0.1321	0.4154	0.083*	
C20B	0.15986 (14)	0.0313 (3)	0.30453 (9)	0.0454 (4)	
H20D	0.0965	0.1142	0.2906	0.068*	
H20E	0.1511	−0.0107	0.3445	0.068*	
H20F	0.1591	−0.0756	0.2782	0.068*	

### Atomic displacement parameters (Å^2^)

**Table:**

	*U*^11^	*U*^22^	*U*^33^	*U*^12^	*U*^13^	*U*^23^
O1A	0.0549 (8)	0.0457 (9)	0.0638 (9)	−0.0041 (8)	0.0218 (7)	−0.0016 (8)
O2A	0.0815 (11)	0.0496 (10)	0.0990 (13)	−0.0137 (10)	0.0331 (11)	−0.0231 (11)
C1A	0.0553 (10)	0.0372 (10)	0.0509 (10)	−0.0109 (10)	0.0145 (8)	0.0010 (9)
C2A	0.0703 (13)	0.0434 (12)	0.0564 (11)	−0.0039 (11)	0.0137 (10)	0.0121 (11)
C3A	0.0581 (11)	0.0529 (13)	0.0469 (10)	0.0006 (11)	0.0058 (8)	0.0020 (11)
C4A	0.0439 (9)	0.0376 (10)	0.0517 (10)	0.0007 (9)	0.0021 (8)	−0.0016 (10)
C5A	0.0340 (7)	0.0274 (8)	0.0473 (9)	0.0018 (7)	0.0066 (6)	−0.0033 (8)
C6A	0.0479 (9)	0.0310 (10)	0.0577 (11)	−0.0080 (8)	0.0067 (8)	−0.0015 (9)
C7A	0.0602 (11)	0.0268 (9)	0.0532 (10)	−0.0034 (9)	0.0099 (8)	0.0031 (9)
C8A	0.0390 (8)	0.0295 (9)	0.0491 (9)	0.0024 (8)	0.0073 (7)	0.0006 (8)
C9A	0.0303 (7)	0.0331 (9)	0.0471 (9)	−0.0006 (7)	0.0102 (6)	−0.0030 (8)
C10A	0.0343 (7)	0.0273 (8)	0.0455 (9)	0.0001 (7)	0.0113 (6)	−0.0024 (8)
C11A	0.0437 (9)	0.0339 (9)	0.0507 (9)	0.0014 (8)	0.0069 (7)	0.0024 (9)
C12A	0.0379 (8)	0.0448 (12)	0.0456 (9)	0.0032 (9)	0.0001 (7)	−0.0011 (9)
C13A	0.0351 (8)	0.0549 (13)	0.0581 (11)	−0.0048 (9)	0.0045 (8)	−0.0099 (11)
C14A	0.0430 (9)	0.0434 (11)	0.0521 (10)	−0.0132 (9)	0.0140 (8)	−0.0035 (10)
C15A	0.0402 (8)	0.0411 (10)	0.0412 (8)	−0.0031 (8)	0.0041 (7)	−0.0015 (9)
C16A	0.0555 (12)	0.0631 (16)	0.0754 (14)	−0.0099 (12)	0.0068 (10)	−0.0270 (14)
C17A	0.0615 (12)	0.0682 (17)	0.0641 (13)	0.0125 (13)	−0.0117 (10)	0.0059 (13)
C18A	0.0765 (14)	0.0499 (13)	0.0617 (13)	−0.0019 (13)	−0.0064 (11)	−0.0132 (12)
C19A	0.0420 (9)	0.0623 (15)	0.0706 (13)	0.0028 (10)	−0.0044 (9)	0.0069 (13)
C20A	0.0431 (9)	0.0330 (10)	0.0620 (11)	0.0074 (8)	0.0122 (8)	−0.0042 (9)
O1B	0.0411 (6)	0.0602 (9)	0.0366 (6)	0.0013 (7)	0.0030 (5)	0.0069 (7)
O2B	0.0763 (10)	0.0602 (11)	0.0493 (8)	−0.0274 (9)	0.0203 (7)	−0.0137 (8)
C1B	0.0599 (11)	0.0365 (10)	0.0416 (9)	0.0044 (9)	0.0061 (8)	−0.0042 (9)
C2B	0.0817 (14)	0.0440 (12)	0.0420 (10)	0.0066 (12)	0.0118 (9)	−0.0094 (10)
C3B	0.0639 (11)	0.0495 (12)	0.0391 (9)	−0.0073 (11)	0.0011 (8)	0.0005 (10)
C4B	0.0418 (8)	0.0426 (10)	0.0393 (8)	0.0012 (8)	0.0039 (7)	0.0053 (8)
C5B	0.0291 (6)	0.0368 (9)	0.0379 (8)	0.0017 (7)	0.0043 (6)	0.0018 (8)
C6B	0.0465 (9)	0.0423 (11)	0.0495 (10)	0.0166 (9)	0.0032 (7)	−0.0044 (10)
C7B	0.0431 (9)	0.0617 (14)	0.0445 (9)	0.0198 (10)	0.0056 (7)	−0.0100 (10)
C8B	0.0343 (7)	0.0434 (10)	0.0391 (8)	0.0039 (8)	0.0080 (6)	−0.0028 (8)
C9B	0.0323 (7)	0.0359 (9)	0.0370 (8)	−0.0008 (7)	0.0023 (6)	−0.0029 (7)
C10B	0.0295 (7)	0.0306 (8)	0.0377 (8)	0.0011 (7)	0.0052 (6)	−0.0026 (8)
C11B	0.0408 (8)	0.0483 (11)	0.0392 (8)	0.0044 (9)	0.0080 (7)	−0.0071 (9)
C12B	0.0410 (8)	0.0448 (10)	0.0335 (8)	−0.0022 (9)	0.0024 (6)	0.0000 (9)
C13B	0.0420 (9)	0.0459 (11)	0.0427 (9)	0.0100 (9)	−0.0029 (7)	−0.0031 (9)
C14B	0.0501 (9)	0.0367 (10)	0.0449 (9)	0.0058 (9)	−0.0050 (7)	−0.0031 (9)
C15B	0.0417 (8)	0.0467 (11)	0.0299 (7)	−0.0012 (9)	0.0038 (6)	0.0006 (8)
C16B	0.0541 (10)	0.0497 (12)	0.0341 (8)	−0.0093 (10)	0.0033 (7)	0.0000 (9)
C17B	0.0593 (11)	0.0640 (16)	0.0513 (11)	−0.0183 (12)	0.0012 (9)	0.0080 (12)
C18B	0.0501 (10)	0.0829 (19)	0.0513 (11)	0.0128 (12)	−0.0024 (9)	0.0138 (13)
C19B	0.0698 (13)	0.0454 (12)	0.0528 (11)	−0.0068 (11)	0.0136 (10)	0.0087 (10)
C20B	0.0308 (7)	0.0492 (11)	0.0570 (10)	−0.0023 (9)	0.0082 (7)	0.0020 (10)

### Geometric parameters (Å, °)

**Table:**

O1A—C15A	1.439 (2)	O1B—C15B	1.437 (2)
O1A—H1OA	0.79 (3)	O1B—H1OC	0.76 (2)
O2A—C16A	1.417 (3)	O2B—C16B	1.412 (3)
O2A—H1OB	0.84 (3)	O2B—H1OD	0.77 (3)
C1A—C2A	1.524 (3)	C1B—C2B	1.525 (3)
C1A—C10A	1.538 (2)	C1B—C10B	1.541 (3)
C1A—H1AA	0.9700	C1B—H1BA	0.9700
C1A—H1AB	0.9700	C1B—H1BB	0.9700
C2A—C3A	1.516 (3)	C2B—C3B	1.516 (3)
C2A—H2AA	0.9700	C2B—H2BA	0.9700
C2A—H2AB	0.9700	C2B—H2BB	0.9700
C3A—C4A	1.532 (3)	C3B—C4B	1.533 (3)
C3A—H3AA	0.9700	C3B—H3BA	0.9700
C3A—H3AB	0.9700	C3B—H3BB	0.9700
C4A—C18A	1.537 (3)	C4B—C19B	1.530 (3)
C4A—C19A	1.541 (3)	C4B—C18B	1.538 (3)
C4A—C5A	1.564 (3)	C4B—C5B	1.561 (2)
C5A—C6A	1.528 (3)	C5B—C6B	1.528 (3)
C5A—C10A	1.556 (2)	C5B—C10B	1.561 (2)
C5A—H5AA	0.9800	C5B—H5BA	0.9800
C6A—C7A	1.528 (3)	C6B—C7B	1.530 (3)
C6A—H6AA	0.9700	C6B—H6BA	0.9700
C6A—H6AB	0.9700	C6B—H6BB	0.9700
C7A—C8A	1.505 (3)	C7B—C8B	1.503 (3)
C7A—H7AA	0.9700	C7B—H7BA	0.9700
C7A—H7AB	0.9700	C7B—H7BB	0.9700
C8A—C11A	1.326 (3)	C8B—C11B	1.328 (2)
C8A—C9A	1.516 (2)	C8B—C9B	1.515 (3)
C9A—C14A	1.531 (3)	C9B—C14B	1.547 (3)
C9A—C10A	1.564 (2)	C9B—C10B	1.570 (2)
C9A—H9AA	0.9800	C9B—H9BA	0.9800
C10A—C20A	1.543 (2)	C10B—C20B	1.533 (2)
C11A—C12A	1.514 (3)	C11B—C12B	1.510 (3)
C11A—H11A	0.9300	C11B—H11B	0.9300
C12A—C17A	1.533 (3)	C12B—C17B	1.536 (3)
C12A—C15A	1.542 (3)	C12B—C13B	1.536 (2)
C12A—C13A	1.545 (3)	C12B—C15B	1.549 (3)
C13A—C14A	1.520 (3)	C13B—C14B	1.532 (3)
C13A—H13A	0.9700	C13B—H13C	0.9700
C13A—H13B	0.9700	C13B—H13D	0.9700
C14A—H14A	0.9700	C14B—H14C	0.9700
C14A—H14B	0.9700	C14B—H14D	0.9700
C15A—C16A	1.511 (3)	C15B—C16B	1.514 (3)
C15A—H15A	0.9800	C15B—H15B	0.9800
C16A—H16A	0.9700	C16B—H16C	0.9700
C16A—H16B	0.9700	C16B—H16D	0.9700
C17A—H17A	0.9600	C17B—H17D	0.9600
C17A—H17B	0.9600	C17B—H17E	0.9600
C17A—H17C	0.9600	C17B—H17F	0.9600
C18A—H18A	0.9600	C18B—H18D	0.9600
C18A—H18B	0.9600	C18B—H18E	0.9600
C18A—H18C	0.9600	C18B—H18F	0.9600
C19A—H19A	0.9600	C19B—H19D	0.9600
C19A—H19B	0.9600	C19B—H19E	0.9600
C19A—H19C	0.9600	C19B—H19F	0.9600
C20A—H20A	0.9600	C20B—H20D	0.9600
C20A—H20B	0.9600	C20B—H20E	0.9600
C20A—H20C	0.9600	C20B—H20F	0.9600
			
C15A—O1A—H1OA	109 (2)	C15B—O1B—H1OC	110.8 (19)
C16A—O2A—H1OB	108 (3)	C16B—O2B—H1OD	108 (2)
C2A—C1A—C10A	113.59 (16)	C2B—C1B—C10B	113.41 (17)
C2A—C1A—H1AA	108.8	C2B—C1B—H1BA	108.9
C10A—C1A—H1AA	108.8	C10B—C1B—H1BA	108.9
C2A—C1A—H1AB	108.8	C2B—C1B—H1BB	108.9
C10A—C1A—H1AB	108.8	C10B—C1B—H1BB	108.9
H1AA—C1A—H1AB	107.7	H1BA—C1B—H1BB	107.7
C3A—C2A—C1A	110.60 (19)	C3B—C2B—C1B	110.31 (17)
C3A—C2A—H2AA	109.5	C3B—C2B—H2BA	109.6
C1A—C2A—H2AA	109.5	C1B—C2B—H2BA	109.6
C3A—C2A—H2AB	109.5	C3B—C2B—H2BB	109.6
C1A—C2A—H2AB	109.5	C1B—C2B—H2BB	109.6
H2AA—C2A—H2AB	108.1	H2BA—C2B—H2BB	108.1
C2A—C3A—C4A	113.19 (17)	C2B—C3B—C4B	114.15 (17)
C2A—C3A—H3AA	108.9	C2B—C3B—H3BA	108.7
C4A—C3A—H3AA	108.9	C4B—C3B—H3BA	108.7
C2A—C3A—H3AB	108.9	C2B—C3B—H3BB	108.7
C4A—C3A—H3AB	108.9	C4B—C3B—H3BB	108.7
H3AA—C3A—H3AB	107.8	H3BA—C3B—H3BB	107.6
C3A—C4A—C18A	107.15 (17)	C19B—C4B—C3B	109.71 (16)
C3A—C4A—C19A	110.44 (18)	C19B—C4B—C18B	107.55 (19)
C18A—C4A—C19A	107.15 (19)	C3B—C4B—C18B	107.29 (18)
C3A—C4A—C5A	108.64 (15)	C19B—C4B—C5B	114.69 (16)
C18A—C4A—C5A	108.61 (17)	C3B—C4B—C5B	108.75 (17)
C19A—C4A—C5A	114.58 (16)	C18B—C4B—C5B	108.59 (14)
C6A—C5A—C10A	110.52 (14)	C6B—C5B—C4B	113.92 (16)
C6A—C5A—C4A	114.37 (15)	C6B—C5B—C10B	110.64 (13)
C10A—C5A—C4A	116.62 (16)	C4B—C5B—C10B	117.31 (13)
C6A—C5A—H5AA	104.6	C6B—C5B—H5BA	104.5
C10A—C5A—H5AA	104.6	C4B—C5B—H5BA	104.5
C4A—C5A—H5AA	104.6	C10B—C5B—H5BA	104.5
C7A—C6A—C5A	110.60 (15)	C5B—C6B—C7B	110.01 (18)
C7A—C6A—H6AA	109.5	C5B—C6B—H6BA	109.7
C5A—C6A—H6AA	109.5	C7B—C6B—H6BA	109.7
C7A—C6A—H6AB	109.5	C5B—C6B—H6BB	109.7
C5A—C6A—H6AB	109.5	C7B—C6B—H6BB	109.7
H6AA—C6A—H6AB	108.1	H6BA—C6B—H6BB	108.2
C8A—C7A—C6A	113.24 (17)	C8B—C7B—C6B	111.98 (14)
C8A—C7A—H7AA	108.9	C8B—C7B—H7BA	109.2
C6A—C7A—H7AA	108.9	C6B—C7B—H7BA	109.2
C8A—C7A—H7AB	108.9	C8B—C7B—H7BB	109.2
C6A—C7A—H7AB	108.9	C6B—C7B—H7BB	109.2
H7AA—C7A—H7AB	107.7	H7BA—C7B—H7BB	107.9
C11A—C8A—C7A	122.23 (18)	C11B—C8B—C7B	121.33 (18)
C11A—C8A—C9A	123.47 (18)	C11B—C8B—C9B	123.32 (17)
C7A—C8A—C9A	114.20 (15)	C7B—C8B—C9B	115.28 (15)
C8A—C9A—C14A	112.30 (14)	C8B—C9B—C14B	112.43 (14)
C8A—C9A—C10A	111.82 (13)	C8B—C9B—C10B	112.11 (15)
C14A—C9A—C10A	113.74 (16)	C14B—C9B—C10B	115.59 (13)
C8A—C9A—H9AA	106.1	C8B—C9B—H9BA	105.2
C14A—C9A—H9AA	106.1	C14B—C9B—H9BA	105.2
C10A—C9A—H9AA	106.1	C10B—C9B—H9BA	105.2
C1A—C10A—C20A	110.07 (16)	C20B—C10B—C1B	109.83 (14)
C1A—C10A—C5A	109.12 (14)	C20B—C10B—C5B	114.16 (15)
C20A—C10A—C5A	112.87 (14)	C1B—C10B—C5B	107.78 (14)
C1A—C10A—C9A	108.45 (13)	C20B—C10B—C9B	109.23 (13)
C20A—C10A—C9A	109.19 (14)	C1B—C10B—C9B	108.46 (15)
C5A—C10A—C9A	107.01 (14)	C5B—C10B—C9B	107.21 (12)
C8A—C11A—C12A	125.74 (18)	C8B—C11B—C12B	125.76 (18)
C8A—C11A—H11A	117.1	C8B—C11B—H11B	117.1
C12A—C11A—H11A	117.1	C12B—C11B—H11B	117.1
C11A—C12A—C17A	108.28 (19)	C11B—C12B—C17B	109.18 (15)
C11A—C12A—C15A	110.18 (14)	C11B—C12B—C13B	108.25 (14)
C17A—C12A—C15A	111.15 (17)	C17B—C12B—C13B	111.03 (19)
C11A—C12A—C13A	107.27 (15)	C11B—C12B—C15B	108.32 (17)
C17A—C12A—C13A	109.46 (16)	C17B—C12B—C15B	107.96 (14)
C15A—C12A—C13A	110.40 (17)	C13B—C12B—C15B	112.04 (14)
C14A—C13A—C12A	112.70 (14)	C14B—C13B—C12B	111.87 (14)
C14A—C13A—H13A	109.1	C14B—C13B—H13C	109.2
C12A—C13A—H13A	109.1	C12B—C13B—H13C	109.2
C14A—C13A—H13B	109.1	C14B—C13B—H13D	109.2
C12A—C13A—H13B	109.1	C12B—C13B—H13D	109.2
H13A—C13A—H13B	107.8	H13C—C13B—H13D	107.9
C13A—C14A—C9A	112.39 (18)	C13B—C14B—C9B	114.45 (17)
C13A—C14A—H14A	109.1	C13B—C14B—H14C	108.6
C9A—C14A—H14A	109.1	C9B—C14B—H14C	108.6
C13A—C14A—H14B	109.1	C13B—C14B—H14D	108.6
C9A—C14A—H14B	109.1	C9B—C14B—H14D	108.6
H14A—C14A—H14B	107.9	H14C—C14B—H14D	107.6
O1A—C15A—C16A	109.64 (16)	O1B—C15B—C16B	107.45 (14)
O1A—C15A—C12A	108.71 (17)	O1B—C15B—C12B	111.38 (17)
C16A—C15A—C12A	114.79 (16)	C16B—C15B—C12B	115.05 (13)
O1A—C15A—H15A	107.8	O1B—C15B—H15B	107.6
C16A—C15A—H15A	107.8	C16B—C15B—H15B	107.6
C12A—C15A—H15A	107.8	C12B—C15B—H15B	107.6
O2A—C16A—C15A	113.41 (19)	O2B—C16B—C15B	108.37 (14)
O2A—C16A—H16A	108.9	O2B—C16B—H16C	110.0
C15A—C16A—H16A	108.9	C15B—C16B—H16C	110.0
O2A—C16A—H16B	108.9	O2B—C16B—H16D	110.0
C15A—C16A—H16B	108.9	C15B—C16B—H16D	110.0
H16A—C16A—H16B	107.7	H16C—C16B—H16D	108.4
C12A—C17A—H17A	109.5	C12B—C17B—H17D	109.5
C12A—C17A—H17B	109.5	C12B—C17B—H17E	109.5
H17A—C17A—H17B	109.5	H17D—C17B—H17E	109.5
C12A—C17A—H17C	109.5	C12B—C17B—H17F	109.5
H17A—C17A—H17C	109.5	H17D—C17B—H17F	109.5
H17B—C17A—H17C	109.5	H17E—C17B—H17F	109.5
C4A—C18A—H18A	109.5	C4B—C18B—H18D	109.5
C4A—C18A—H18B	109.5	C4B—C18B—H18E	109.5
H18A—C18A—H18B	109.5	H18D—C18B—H18E	109.5
C4A—C18A—H18C	109.5	C4B—C18B—H18F	109.5
H18A—C18A—H18C	109.5	H18D—C18B—H18F	109.5
H18B—C18A—H18C	109.5	H18E—C18B—H18F	109.5
C4A—C19A—H19A	109.5	C4B—C19B—H19D	109.5
C4A—C19A—H19B	109.5	C4B—C19B—H19E	109.5
H19A—C19A—H19B	109.5	H19D—C19B—H19E	109.5
C4A—C19A—H19C	109.5	C4B—C19B—H19F	109.5
H19A—C19A—H19C	109.5	H19D—C19B—H19F	109.5
H19B—C19A—H19C	109.5	H19E—C19B—H19F	109.5
C10A—C20A—H20A	109.5	C10B—C20B—H20D	109.5
C10A—C20A—H20B	109.5	C10B—C20B—H20E	109.5
H20A—C20A—H20B	109.5	H20D—C20B—H20E	109.5
C10A—C20A—H20C	109.5	C10B—C20B—H20F	109.5
H20A—C20A—H20C	109.5	H20D—C20B—H20F	109.5
H20B—C20A—H20C	109.5	H20E—C20B—H20F	109.5
			
C10A—C1A—C2A—C3A	57.5 (2)	C10B—C1B—C2B—C3B	58.9 (2)
C1A—C2A—C3A—C4A	−58.9 (2)	C1B—C2B—C3B—C4B	−57.5 (3)
C2A—C3A—C4A—C18A	170.75 (19)	C2B—C3B—C4B—C19B	−75.1 (2)
C2A—C3A—C4A—C19A	−72.9 (2)	C2B—C3B—C4B—C18B	168.35 (18)
C2A—C3A—C4A—C5A	53.6 (2)	C2B—C3B—C4B—C5B	51.1 (2)
C3A—C4A—C5A—C6A	179.29 (16)	C19B—C4B—C5B—C6B	−56.8 (2)
C18A—C4A—C5A—C6A	63.1 (2)	C3B—C4B—C5B—C6B	179.95 (15)
C19A—C4A—C5A—C6A	−56.7 (2)	C18B—C4B—C5B—C6B	63.5 (2)
C3A—C4A—C5A—C10A	−49.6 (2)	C19B—C4B—C5B—C10B	74.7 (2)
C18A—C4A—C5A—C10A	−165.82 (16)	C3B—C4B—C5B—C10B	−48.5 (2)
C19A—C4A—C5A—C10A	74.4 (2)	C18B—C4B—C5B—C10B	−164.97 (18)
C10A—C5A—C6A—C7A	59.7 (2)	C4B—C5B—C6B—C7B	−163.57 (14)
C4A—C5A—C6A—C7A	−166.33 (16)	C10B—C5B—C6B—C7B	61.74 (19)
C5A—C6A—C7A—C8A	−51.7 (2)	C5B—C6B—C7B—C8B	−54.2 (2)
C6A—C7A—C8A—C11A	−135.30 (19)	C6B—C7B—C8B—C11B	−133.6 (2)
C6A—C7A—C8A—C9A	48.2 (2)	C6B—C7B—C8B—C9B	49.5 (2)
C11A—C8A—C9A—C14A	2.8 (2)	C11B—C8B—C9B—C14B	0.7 (3)
C7A—C8A—C9A—C14A	179.18 (15)	C7B—C8B—C9B—C14B	177.58 (16)
C11A—C8A—C9A—C10A	132.04 (19)	C11B—C8B—C9B—C10B	132.89 (18)
C7A—C8A—C9A—C10A	−51.6 (2)	C7B—C8B—C9B—C10B	−50.2 (2)
C2A—C1A—C10A—C20A	73.3 (2)	C2B—C1B—C10B—C20B	71.4 (2)
C2A—C1A—C10A—C5A	−51.1 (2)	C2B—C1B—C10B—C5B	−53.5 (2)
C2A—C1A—C10A—C9A	−167.33 (18)	C2B—C1B—C10B—C9B	−169.27 (16)
C6A—C5A—C10A—C1A	−178.74 (15)	C6B—C5B—C10B—C20B	60.38 (19)
C4A—C5A—C10A—C1A	48.38 (19)	C4B—C5B—C10B—C20B	−72.6 (2)
C6A—C5A—C10A—C20A	58.6 (2)	C6B—C5B—C10B—C1B	−177.32 (15)
C4A—C5A—C10A—C20A	−74.33 (19)	C4B—C5B—C10B—C1B	49.7 (2)
C6A—C5A—C10A—C9A	−61.59 (17)	C6B—C5B—C10B—C9B	−60.74 (18)
C4A—C5A—C10A—C9A	165.53 (13)	C4B—C5B—C10B—C9B	166.26 (16)
C8A—C9A—C10A—C1A	174.49 (15)	C8B—C9B—C10B—C20B	−70.25 (18)
C14A—C9A—C10A—C1A	−57.00 (18)	C14B—C9B—C10B—C20B	60.4 (2)
C8A—C9A—C10A—C20A	−65.57 (19)	C8B—C9B—C10B—C1B	170.06 (14)
C14A—C9A—C10A—C20A	62.94 (18)	C14B—C9B—C10B—C1B	−59.31 (19)
C8A—C9A—C10A—C5A	56.90 (17)	C8B—C9B—C10B—C5B	53.94 (17)
C14A—C9A—C10A—C5A	−174.59 (13)	C14B—C9B—C10B—C5B	−175.43 (15)
C7A—C8A—C11A—C12A	−171.68 (17)	C7B—C8B—C11B—C12B	−179.05 (18)
C9A—C8A—C11A—C12A	4.4 (3)	C9B—C8B—C11B—C12B	−2.3 (3)
C8A—C11A—C12A—C17A	137.0 (2)	C8B—C11B—C12B—C17B	97.3 (2)
C8A—C11A—C12A—C15A	−101.3 (2)	C8B—C11B—C12B—C13B	−23.7 (3)
C8A—C11A—C12A—C13A	19.0 (3)	C8B—C11B—C12B—C15B	−145.38 (19)
C11A—C12A—C13A—C14A	−49.8 (2)	C11B—C12B—C13B—C14B	50.4 (2)
C17A—C12A—C13A—C14A	−167.1 (2)	C17B—C12B—C13B—C14B	−69.4 (2)
C15A—C12A—C13A—C14A	70.3 (2)	C15B—C12B—C13B—C14B	169.82 (17)
C12A—C13A—C14A—C9A	59.8 (2)	C12B—C13B—C14B—C9B	−55.3 (2)
C8A—C9A—C14A—C13A	−33.8 (2)	C8B—C9B—C14B—C13B	27.9 (2)
C10A—C9A—C14A—C13A	−162.06 (14)	C10B—C9B—C14B—C13B	−102.53 (18)
C11A—C12A—C15A—O1A	−47.6 (2)	C11B—C12B—C15B—O1B	−176.96 (13)
C17A—C12A—C15A—O1A	72.4 (2)	C17B—C12B—C15B—O1B	−58.86 (19)
C13A—C12A—C15A—O1A	−165.91 (15)	C13B—C12B—C15B—O1B	63.70 (19)
C11A—C12A—C15A—C16A	−170.79 (18)	C11B—C12B—C15B—C16B	60.46 (19)
C17A—C12A—C15A—C16A	−50.8 (3)	C17B—C12B—C15B—C16B	178.56 (18)
C13A—C12A—C15A—C16A	70.9 (2)	C13B—C12B—C15B—C16B	−58.9 (2)
O1A—C15A—C16A—O2A	75.0 (3)	O1B—C15B—C16B—O2B	73.8 (2)
C12A—C15A—C16A—O2A	−162.28 (18)	C12B—C15B—C16B—O2B	−161.55 (17)

### Hydrogen-bond geometry (Å, °)

**Table:**

*D*—H···*A*	*D*—H	H···*A*	*D*···*A*	*D*—H···*A*
O1A—H1OA···O1B^i^	0.78 (3)	2.06 (3)	2.839 (2)	174 (3)
O2A—H1OB···O1B^i^	0.84 (4)	2.14 (4)	2.931 (2)	158 (3)
O1B—H1OC···O2B^ii^	0.76 (2)	1.96 (2)	2.719 (2)	178 (3)
O2B—H1OD···O1A^iii^	0.77 (3)	2.13 (3)	2.795 (3)	145 (3)

Symmetry codes: (i) *x*+1, *y*, *z*; (ii) −*x*, *y*+1/2, −*z*; (iii) *x*−1, *y*−1, *z*.
